# ‘The agency of observation not to be neglected’: complementarity, causality and the arrow of events in quantum and quantum-like theories

**DOI:** 10.1098/rsta.2022.0295

**Published:** 2023-10-02

**Authors:** Arkady Plotnitsky

**Affiliations:** Literature, Theory, Cultural Studies Program; Philosophy and Literature Program, Purdue University, West Lafayette, IN 47907, USA

**Keywords:** arrow of events, complementarity, causality, probability, quantum-like theories, reality without realism (RWR)

## Abstract

The argument of this article is grounded in the irreducible interference of observational instruments in our interactions with nature in quantum physics and, thus, in the constitution of quantum phenomena versus classical physics, where this interference can, in principle, be disregarded. The irreducible character of this interference was seen by N. Bohr as the principal distinction between classical and quantum physics and grounded his interpretation of quantum phenomena and quantum theory. Bohr saw complementarity as a generalization of the classical ideal of causality, which defined classical physics and relativity. While intimated by Bohr, the relationships among observational technology, complementarity, causality and the arrow of events (a new concept that replaces the arrow of time commonly used in this context) were not addressed by him either. The article introduces another new concept, that of quantum causality, as a form of probabilistic causality. The argument of the article is based on a particular interpretation of quantum phenomena and quantum theory, defined by the concept of ‘reality without realism (RWR)’. This interpretation follows Bohr's interpretation but contains certain additional features, in particular the Dirac postulate. The article also considers quantum-like (Q-L) theories (based in the mathematics of QM) from the perspective it develops.

This article is part of the theme issue ‘Thermodynamics 2.0: Bridging the natural and social sciences (Part 2)’.

## 1. Introduction

The argument of this article is grounded in the irreducible interference of observational instruments in our interactions with nature in quantum physics and, thus, in the constitution of quantum phenomena versus classical physics or relativity, where this interference can be disregarded, at least in principle. The irreducible character of this interference was seen by N. Bohr as the principal distinction between classical and quantum physics and has grounded his interpretation of quantum phenomena and quantum theory, specifically quantum mechanics (QM) and quantum field theory (QFT), developed by him in conjunction with the concepts of complementarity and (quantum) phenomenon. In this view, an observation in quantum physics, become a singular, each time unique, act of creation of a quantum phenomenon, rather than an observation of the pre-existing property of the quantum object considered. Bohr saw complementarity as a generalization of the concept of classical causality, which defined classical physics or relativity. This generalization, not explained by Bohr, will be understood here by means of the concept of quantum causality in conjunction with a new concept, the arrow of events, replacing that of the arrow of time, commonly used in this context. While shaping Bohr's argumentation, the relationships among observational technology, complementarity, causality and the arrow of events were not expressly discussed by him either. This article argues, however, that these relationships are essential in quantum theory.

The argument of the article is based on a particular interpretation of quantum phenomena and quantum theory, specifically QM and QFT. This article will only be concerned with QM and QFT in their currently standard forms, and will only mention alternative quantum theories, such as Bohmian mechanics, in passing. Quantum phenomena will be assumed to be defined by the fact that in considering them the Planck constant, *h*, must be taken into account.^1^ The present interpretation follows Bohr's ultimate interpretation, developed by Bohr in the late 1930s and based, in addition to complementarity, introduced in 1927, on a new concept, that of (quantum) phenomenon. Both interpretations belong to the class of interpretations based on the concept of reality without realism (RWR) and designated here as RWR interpretations.^2^ In accordance with this concept, these interpretations place the emergence of quantum phenomena beyond representation and knowledge or even conception, to be designated as respectively *weak* and *strong* RWR interpretations. This article adopts a strong RWR interpretation, as did Bohr in the ultimate version of his interpretation. In these interpretations, the capacity of the mathematics of QM or QFT to predict the outcomes of quantum experiments is beyond conception as well. We know *how* this mathematics works, but we do not and possibly cannot know or even conceive of *why* it works. These predictions are in general probabilistic, regardless of the quantum objects considered, no matter how elementary. This is, however, fully in accord with all experimental evidence available thus far, which only allows for such predictions. The present interpretation contains additional, epistemologically more radical, features, not found in Bohr's interpretation. Most especially, the concept of a quantum object is assumed to be applicable only at the time of observation, rather than, as in Bohr, to something existing independently. I shall designate this assumption ‘the Dirac postulate’, for the reason explained below.

It would be difficult to argue that quantum phenomena or QM or QFT require RWR interpretations, or the Dirac postulate, and it is not my aim to do so. I only claim the logical consistency of these interpretations and their accord with the experimental evidence, *as currently constituted*, a qualification assumed throughout this article. New evidence can make any theory or interpretation obsolete.

The next section outlines RWR interpretations of quantum phenomena and QM. Section 3 considers the concepts of quantum causality and the arrow of events in quantum physics. Section 4 discusses quantum-like (Q-L) theories, based in the mathematics of QM but used elsewhere, from the perspective developed in the earlier sections.

## 2. Observation as creation: quantum phenomena and reality without realism

The concept of RWR, which grounds RWR interpretations of quantum phenomena and QM, is a specific and epistemologically radical concept. But it presupposes general concepts of reality and existence, assumed to be primitive concepts and not given analytical definitions. By ‘reality’ I refer to that which is assumed to exist without making any claims concerning the *character* of this existence, claims that define realism. The absence of such claims allows one to place this character beyond representation or even conception, which placement defines the concept of RWR. I understand existence as a capacity to have effects on the world. Although this is not always realized, the assumption that something is real, including of the RWR-type, is inevitably inferred from such effects.

It may be useful to begin this section by explaining realism, to which RWR thinking is juxtaposed here. As a mode of scientific or philosophical thought, realism is manifested in the corresponding theories, commonly representational in character. Such theories aim to represent the reality they consider, in modern, post-Galilean, physics by mathematized models suitably idealizing this reality. It is also possible to assume an independent structure (defined by properties and relationships among them) of the reality considered, while admitting that it is either (A) not possible to represent this architecture or (B) to form a rigorously specified concept of it, either at a given point in history or even ever. Under (A), a theory that is merely predictive could be accepted for lack of a realist alternative, usually with the hope that a future theory will do better by being a representational theory. Einstein adopted this attitude toward QM. What, then, grounds realism most fundamentally is the assumption that the ultimate constitution of reality possesses properties and the relationships between them, or, as in (ontic) structural realism, just a structure, the more elemental constituents of which are not defined in terms of properties [5]. Such properties, relationships, or structures may either be ideally represented or be unrepresentable or unknown, or even unknowable, but they are still assumed to be conceivable, commonly with a hope that they will be eventually so represented.

Thus, classical mechanics (used in dealing with individual objects and small systems), classical statistical mechanics (used in dealing with large classical systems), chaos theory (used in dealing with classical systems that exhibit a highly nonlinear behaviour) and relativity, special and general, are realist theories, offering mathematically idealized representations of the physical reality considered. All these theories are also based on the assumption that we can observe the phenomena considered without appreciably disturbing them, and as a result, identify them with the corresponding objects and their independent behaviour. It is this assumption that makes these theories realist because it allows ideally to represent this behaviour and to predict it, either ideally exactly or, as in classical statistical physics or chaos theory, probabilistically, by using this representation. Not all realist theories or interpretations are of this type, including in the case of realist interpretations of QM, which are possible. On the other hand, this *identification* is no longer possible in dealing with quantum phenomena, regardless of interpretation (although realism is, again, possible), because of the irreducible role of measuring instruments in the constitution of quantum phenomena, a feature that grounded Bohr's interpretation in all its versions. Bohr adjusted, sometimes significantly, his interpretation. This requires one to specify to which version of his interpretation one refers, which I shall do, while focusing on his ultimate, strong RWR, interpretation.^3^ As, however, Bohr argued already in the so-called Como lecture of 1927, which presented his first interpretation of QM, in classical physics and relativity ‘our … description of physical phenomena [is] based of the idea that the phenomena concerned may be observed *without disturbing them appreciably*’ [6] (v. 1, p. 53; emphasis added). Bohr is careful to refer to the idea and hence the assumption rather than saying that such is in fact the case. This assumption is, however, workable in these theories for all practical purposes. By contrast, ‘any observation of atomic phenomena will involve an *interaction [of the object under investigation] with the agency of observation* not to be neglected’ [6] (v. 1, p. 54; emphasis added). It is the irreducible nature of this interaction and thus the interference of technology into physical reality in quantum physics that is responsible, via for changing the nature of causality and probability, from classical to quantum, and of the way the arrow of events works in quantum physics. This interaction *gives rise* to a quantum phenomenon rather than *disturbs* the object observed [6] (v. 2, p. 64).

In RWR interpretations, QM does not represent the physical emergence of quantum phenomena, which emergence is placed even beyond conception in strong RWR interpretations, such as Bohr's ultimate interpretation or the interpretation adopted here. These interpretations assume that nothing can be said, by means of QM or otherwise, or even thought about what happens between quantum experiments, which define quantum events or phenomena. This assumption will be designated here as the Heisenberg postulate, because W. Heisenberg was the first to have made it (in its weak form, rather than placing the reality ultimately responsible for quantum phenomena beyond conception altogether) in his discovery of QM. Heisenberg eventually changed his position to a form of mathematical realism, with a Platonist bent. He thought that it may be possible to represent the ultimate reality responsible for quantum phenomena (and hence what happens between quantum experiments) by means of mathematics apart from ordinary language or concepts, or even physical concepts, at least as the latter are conventionally understood, say, in classical physics or relativity, while arguing that our language and concepts could only apply, at the level of observation, to quantum phenomena [7] (pp. 145–189).

Nor, in RWR interpretations, does QM represent quantum phenomena themselves, which are represented by classical physics, the assumption central for Bohr and to be designated as the Bohr postulate, thus completing the trio of the main postulates assumed in this article—the Heisenberg, Bohr and Dirac postulates. In fact, there is a quartet of postulates governing this article if one adds Born's rule, which is in effect the postulate, necessary to connect, in the way explained below, the formalism to the outcomes of measurements (classical by the Bohr postulate) in terms of probabilities. QM only predicts, in general probabilistically, the outcomes of quantum experiments, registered classically, and to do so it requires Born's rule. No other predictions are, again, possible on experimental grounds, because the repetition of the identically prepared quantum experiments in general leads to different outcomes. However, the nature of the probabilities used is different from those of classical physics, even in realist interpretations of QM. These probabilities are non-additive: the joint probability of two or more mutually exclusive alternatives in which an event might occur is not equal to the sum of the probabilities for each alternative, as in classical (Kolmogorovian) probability theory. How does QM calculate these probabilities? Although routine by now, the mathematics of QM was a radical change from all mathematics previously used in physics in the following aspects—the use of complex numbers, C, non-commutativity and Born's rule:

(1) While all previous physics used, *fundamentally*, mathematics over real numbers, R, and was finite-dimensional, QM uses Hilbert spaces over complex numbers, C, which are abstract vector spaces of both finite and infinite dimensions. I qualify because classical physics or relativity may use complex numbers, but (as when using Fourier analysis) only practically for calculations. They do not figure in the final solutions of the equations used or are related to what is observed; and everything that we can observe is represented by real (technically, rational) numbers. QM had to find a different way to connect its formalism to observed quantum phenomena, by using (3).
(2) The second key feature is the non-commutativity of Hilbert space-vectors and especially operators, known as ‘observables’, which are complex *mathematical* quantities, as opposed to classical physics or relativity, where all observable quantities are represented by commuting functions of real variables. These complex quantities are related to *physically* observable real quantities not in terms of representation but by using (3).
(3) Born's rule, in effect a postulate, or an analogous rule (such as von Neumann's projection postulate or Lüder's postulate), establishes the relation between the so-called ‘quantum amplitudes’, which are complex Hilbert-space quantities, and probabilities, which are real numbers. Born's rule does so by using square moduli of these quantities (or, equivalently, by multiplying them by their complex conjugates), which are real quantities. Technically, amplitudes are first linked to probability densities.

The probabilities involved are, again, non-additive: they obey the law of the addition of ‘amplitudes’. These amplitudes, which are, again, complex quantities, are associated with possible alternative events, to the sum of which Born's rule is then applied, given real numbers corresponding to probabilities of these events. I shall only refer to Born's rule from now on. In the simplest case, when ψ is a wave function for a particle in the (position) Hilbert space, Born's rule says that the probability density function *p (x, y, z)* for predicting a measurement of the position at time *t_1_* is equal to $|\psi (x,y,z,t_{1}{|}^{2}$. Integrating over this density gives the probability or (if one repeats the experiment many times) statistics of finding the particle in a given area.^4^

Although Born's or similar rules are connected naturally to the formalism, they are added to rather than contained in it. We do not know why these rules work, but they do. It is, however, difficult to underappreciate the significance of Born's rule. It accomplishes two remarkable things: (1) it connects the formalism (over C) to the outcome of the experiments (which are always encoded in real, technically, rational numbers) and does so strictly in terms of probabilities; and (2) correlatively, it links the quantum to the classical, in accord with the Bohr postulate, with the quantum defined by the Heisenberg postulate, in the present, strong RWR, view. In dealing with individual or simple systems, in classical physics or relativity it is possible to use the formalism, as a suitable mathematical idealization, in describing in a continuous and classically causal way the natural processes considered and thus directly relate such descriptions to the outcomes of the experiments. In other words, such mathematized description or representations directly lead to predictions, in this case ideally exact, deterministic. More complex systems may require the recourse probability, but, in contrast to QM, not in the essential way, because the ultimate constituents of such systems are still assumed to be treatable deterministically. This is never possible in considering quantum phenomena, no matter how simple the system. Even in realist interpretations (which aim at a continuous and classically causal representation of the reality ultimately responsible for quantum phenomena) QM only, again, no matter how simple the system, *enables us* to predict probabilistically possible future phenomena on the basis of already established phenomena, always discrete relative to each other. But these predictions, while impossible without QM, are not possible without Born's rule either.

The features just outlined do not exclude a realist or classically causal theory of quantum phenomena or a realist or classical causal interpretation of QM. They and hence quantum phenomena only exclude deterministic interpretations of QM, interpretations that allow for (ideally) exact predictions concerning the behaviour of individual quantum systems. However, because of the irreducible role of observational instruments in the constitution of quantum phenomena, RWR interpretations preclude realism and, as a consequence, classical causality, rather than only determinism. I explain classical causality below. Briefly, it is defined by the claim that the state, *X*, of a physical system is determined, in accordance with a law, at all future moments of time once its state, *A*, is determined at a given moment of time. Determinism is defined here by our capacity to make exact predictions, which are not guaranteed in the presence of classical causality, as in classical statistical physics or chaos theory.

Eventually, Bohr adopted the term ‘phenomenon’ as applicable strictly to what is observed in measuring instruments:

I advocated the application of the word phenomenon exclusively to refer to *the observations obtained under specified circumstances*, including an account of the whole experimental arrangement. In such terminology, the observational problem is free of any special intricacy since, in actual experiments, all observations are expressed by unambiguous statements referring, for instance, to the registration of the point at which an electron arrives at a photographic plate. Moreover, speaking in such a way is just suited to emphasize that the appropriate physical interpretation of the symbolic quantum-mechanical formalism amounts only to predictions, of determinate or statistical character, pertaining to individual phenomena appearing under conditions defined by classical physical concepts [describing the observable parts of instruments]. [6] (v. 2, p. 64; emphasis added)

As defined by ‘*the observations [already] obtained under specified circumstances*’, phenomena only refer to events that have already occurred and not to possible future events predicted by QM. This is the case even if these predictions are ideally exact, which they can be in certain circumstances (without allowing for deterministic predictions concerning the overall behaviour of the system considered), as in the Einstein-Podolsky-Rosen (EPR) type experiments. Bohr's reasons for this view are essentially related to his concept of complementarity, as instantiated in his ultimate, strong RWR, interpretation. As a more general concept (which does not require an RWR interpretation), complementarity is defined by:

(A) a mutual exclusivity of certain phenomena, entities or conceptions; and yet
(B) the possibility of considering each one of them separately at any given point; and
(C) the necessity of considering all of them at different moments of time for a comprehensive account of the totality of phenomena that one must consider in quantum physics.

A paradigmatic example is the mutually exclusivity of the *exact* simultaneous position and momentum measurements in view of the uncertainty relations, Δ*q*Δ*p* ≅ *h* (where *q* is the coordinate, *p* is the momentum in the corresponding direction), which are experimentally confirmed laws independent of any theory, but with which QM is fully in accord. Both variables can be measured simultaneously inexactly. When, however, it comes to exact measurement, one can only perform either one measurement or the other and, in Bohr's ultimate interpretation, define one or the other corresponding phenomenon, but never both together, in accordance with (A). On the other hand, one can always decide and thus has a freedom (at least a sufficient freedom) of choice to perform either measurement, as reflected in (B) and (C).^5^

In Bohr's ultimate, strong RWR, interpretation, complementarity applied strictly to quantum phenomena observed in measuring instruments described by classical physics, and not to quantum objects, which were placed beyond conception. According to Bohr: ‘Evidence obtained under different experimental conditions cannot be comprehended within a single picture, but must be regarded as complementary in the sense that only the totality of the phenomena [some of which are mutually exclusive] exhaust the *possible* information about the objects' [6] (v. 2, p. 40; emphasis added). In classical mechanics, it is possible to comprehend all the information about each object at each moment in time within a single picture because the interference of measurement can be neglected. This assumption allows one to identify the phenomenon and the object under investigation and to establish determinately the quantities defining this information, such as the position and the momentum of each object, in the same experiment. In quantum physics, this interference cannot be neglected. This leads to different experimental conditions for each measurement on a quantum object and their complementarity, in correspondence with the uncertainty relations, which preclude the simultaneous exact measurement of both variables, always in principle possible in classical physics. The situation implies two incompatible pictures of what is observed, as phenomena, in the instruments used. Hence, the *possible* information concerning a quantum object, the information *possibly to be found* in these instruments, could only be exhausted by the mutually incompatible evidence obtainable under different experimental conditions.

On the other hand, once made, either measurement, say, that of the position, will provide the *complete actual* information about the system's state, as complete as possible, at this moment in time. One could never obtain the complementary information, provided by the momentum measurement, at this moment in time, because to do so one would need simultaneously to perform a complementarity experiment on it, which is impossible. By (B), however, one can always decide to perform either one or the other experiment at any given moment in time. Each measurement establishes the only reality there is, and the alternative decision would establish a different reality. Instead of relating to an arbitrarily selection of one or the other parts of a pre-existing physical reality, our decisions concerning which experiment to perform establish the *single* reality that defines what *type* of quantity can be observed or predicted, while precluding the complementary alternative. Hence, parts (B) and (C) of the above definition are as important as part (A) and disregarding them can lead to misunderstandings of Bohr's concept, often misleadingly identified with just (A).

Bohr's complementarity is not only about a mutual exclusivity of certain entities but also about performing quantum experiments and making predictions by human agents, in some of which a mutual exclusivity becomes necessary. That we have a free (or a sufficiently free) choice as concerns what kind of experiment to perform is, as noted by Bohr, in accordance with the very idea of experiment in science, including in classical physics [8] (p. 699). In classical physics or relativity, however, this freedom merely concerns which part of the already established reality one decides to consider. In principle, all variables necessary for defining the future course of reality, in accord with classical causality, can always be determined at any moment in time, as there is no complementarity or the uncertainty relations. By contrast, quantum physics gives this freedom a fundamental role, as reflected in complementarity. By implementing our decision concerning what we want to do, which measurement to perform, we define the character of physical reality and its future course. This decision only allows one to make certain types of predictions and irrevocably excludes certain other, *complementary*, types of possible predictions. Moreover, each new measurement, M_2_, at a later moment in time t_2_, creates, in E. Schrödinger's apt phrase, a new ‘expectation-catalog’, enabled by QM (cum Born's rule) and the data obtained in this measurement, for possible future measurements [9] (p. 154). This new measurement, as a new event, even if one measures the same variable, makes the previous expectation-catalog, defined by a previous measurement, M_1_, at time t_1_ (which could have been used for predicting outcomes of M_2_), meaningless as concerns any prediction after M_2_ is made.

It is true that our decision which measurement to perform only establishes a *possible* future course of reality, because, while we can control the set-up of the experiment, we cannot control the outcome, which fact also gives the measurement objectivity, enabling quantum physics to conform to the requirements of physics as a mathematical-experimental science. Nevertheless, we always have freedom, or, again, a sufficient freedom to make this choice or to change our choice and thus a future course of reality, as opposed to, as in classical physics or relativity, follow what is bound to happen in any event, regardless of our decision of what to do. There, such a decision can only affect what part of a pre-existing reality we would know. By contrast, in quantum physics, it is not a matter of partial knowledge of what is already there, but of creating, by interacting with nature by means of technology, a new reality, shaping a future course of events.

I qualify by ‘a sufficient degree’ throughout because, it is difficult always to know what shapes our decision or changing it at any point. So-called ‘superdeterminism’ denies that we ever do, by assuming that the outcome of all events in the universe is predetermined in advance from the Big Bang on [10]. This view can be put aside here because (its other problems aside) it is realist and classical causal, and hence incompatible with RWR interpretations. There are, however, factors, interior and exterior, that shaped our decision and limit our freedom of choice, which make the category of decision preferable to that of (free) choice. Nevertheless, the reality and its future course thus arising are still defined not only by the pre-existing independent physical reality considered (which does contribute to the initial conditions of the experiment) but by local circumstances, scientific, social, psychological, or other, of a given situation of observation. The role of human decision and freedom of choice, combined with complementarity, in observationally defining reality, has figured significantly in the debates concerning quantum foundations, as in considering the EPR experiment, central to the Bohr-Einstein debate, and then in the Bell, Kochen-Specker and Conway-Kochen (free will) theorems.

The interpretation adopted in this article gives the situation just outlined a more radical character by adopting the Dirac postulate, defined as such in [4] but in effect also used in [1–3]. Both Bohr and the present interpretation assume that the (RWR) reality ultimately responsible for quantum phenomena exists independently, as does nature or matter, in the first place, which amounts to the assumption that it has existed before we existed and will continue to exist when we will no longer exist. On the other hand, while in Bohr's interpretation, the concept of a quantum object, such as an electron or a photon, is associated, on Kantian lines, with the RWR type reality ultimately responsible for quantum phenomena, in the present interpretation, the concept of a quantum object is assumed to be an idealization (of the RWR type) applicable only at the time of observation. This assumption is the Dirac postulate.

At the same time, the present interpretation (or Bohr's interpretation, which is not different in this respect) does not imply a uniform or otherwise unified character of the ultimate, RWR-type, reality considered in QM, a character only manifesting itself differently in quantum experiments. In fact, this assumption would be manifestly in conflict with strong RWR interpretations, which preclude any conception of this reality and, hence, that of its unity or oneness, uniform or not. It is true that, as inconceivable, this reality cannot be assumed to conform to any concept of multiplicity either. It cannot be conceived either as single or as multiple. Accordingly, the situation is as follows. While each time unthinkable, this reality makes each quantum phenomenon, or by the Dirac postulate, each quantum object, as an effect of this reality, singular and unrepeatable, unique. Each quantum phenomenon manifests the inconceivable nature of this reality (or of the corresponding quantum object, which remains beyond conception) each time one encounters this reality through its effects. One can always repeat the set-up of a given measurement, because this set-up can be classically controlled. Not so, however, as concerns the outcomes of thus repeated measurements. Such outcomes are ideally the same and are predictable ideally exactly in classical or relativistic experiments dealing with individual or simple systems. Probability only enters when the systems considered have a great mechanical complexity. By contrast, in identically prepared quantum experiments, the outcomes will in general be different no matter how elementary the quantum objects considered. By ‘identically prepared’ I refer to the states of the observed parts of measuring instruments, which states are repeatable because they are described classically. Observable outcomes of identically prepared quantum experiments are not repeatable.

Could one, then, while assuming the Dirac postulate, still speak of the same quantum object, say, the same electron, in two or more successive observations, such as those defining positions? Each defines an electron, with the same mass, charge and spin, in two different positions at two different moments in time, keeping in mind that in RWR interpretations all these properties are those of observational devices. But is it the same electron? Rigorously, if the concept of a quantum object is only applicable at the time of observation, then a new measurement could only concern a new quantum object, such as a new electron. To consider them as the same electron is, however, a permissible idealization, still statistical in character (because it is not guaranteed that the second observation will register anything at all), in low-energy (QM) regimes. By contrast, speaking of the same electron in successive measurements in high-energy (QFT) regimes is meaningless, because these measurements can register quantum objects of different types, say, in the case quantum electrodynamics (QED) an electron in the initial and a positron or a photon in the next measurement [1] (pp. 279–292). This aspect of QED emerged with the introduction of Dirac's equation for the relativistic (high-energy) electron, revealed to be also that for the positron, which is why I adopt the term ‘the Dirac postulate’. There are, however, reasons, some noted below, to assume that the postulate also applies in low-energy (QM) regimes [3].

Two concepts essential in classical physics and relativity, ‘measurement’ and ‘causality’, become no longer applicable in quantum theory in RWR interpretations. In Bohr's or the present interpretation, a quantum measurement *does not measure* or even *observe* any property of the reality ultimately responsible for quantum phenomena, which this reality is not assumed to possess before or even during the act of observation. By using an instrument (capable of registering quantum phenomena) an observation *creates* a quantum phenomenon by an interaction between this instrument and the quantum object, with the latter concepts only applicable at the time of observation in the present view. Then what is so observed can be measured classically just as one measures what is observed in classical physics. In quantum physics, observations create quantum phenomena, while measurements then classically measure physical properties observed in instruments and not those of quantum objects. It follows that measuring instruments contain both the observable classical parts and unobservable quantum strata, which enable their interactions with quantum objects. This interaction is quantum and thus cannot be observed and, in RWR interpretations, represented or even conceived of.

This concept of quantum measurement, in conjunction with complementarity, leads to the corresponding understanding of the key feature of QM, the non-commutativity of certain quantum variables, such as those associated with the measurements of a momentum, *P*, and a coordinate *Q*, PQ−QP=iℏ (and hence is not zero and PQ≠QP). This formula is also connected to the uncertainty relations, Δ*q*Δ*p* ≅ *h* (where *q* is the coordinate, *p* is the momentum in the corresponding direction), which are part of the experimental confirmation of QM. The uncertainty relations are an experimentally confirmed law, independent of any theory. In Bohr's and the present view, as correlative to complementarity, the uncertainty relations are understood not only as the impossibility of *exactly measuring* both variables simultaneously, but also the impossibility of simultaneously *defining* both. Commonly, non-commutativity is seen as relating to the fact that, if one measures two physical properties involved in one order and then in the other, the outcome would in general be different, which is not the case in classical physics. However, the present understanding of quantum measurement, as first a creation of quantum phenomenon, unique each time, offers a deeper view of this non-commutativity and the difference in the outcomes in reversing the order of measuring complementary variables, as discussed in [1] (pp. 118–122). This view is as follows. It is true that the outcomes will be different, if, in the experiment with the initial preparation of measuring instruments at time t_01_, one makes first the position measurement, M_1Q_, at time t_11_ and then the momentum measurement, M_2P_ at time *t*_21_, and then, with the same initial preparation of measuring instruments (which is possible because one can control the instruments classically), at time *t*_02_ reverse the order of the quantities we measure, by first measuring the momentum, M_1P_, at time *t*_12_ and then the position at time *t*_22_, M_2Q_. As is, however, reflected in my double indexing, each set of measurements happens at a different set of time intervals (*t*_11_ − *t*_01_ = *t*_21_ − *t*_11_ = *t*_21_ − *t*_20_ = *t*_22_ − *t*_21_) and on a different quantum object. One cannot reverse the order of measurements for the same quantum object. As discussed in §4, this situation has important implications for Q-L theories.

The nature of causality in QM changes as well because *classical* causality defining classical physics is no longer possible, at least in RWR interpretations. As noted, by ‘classical causality’ I refer to the claim that the state, *X*, of a physical system is determined, in accordance with a law, at all future moments of time once its state, *A*, is determined at a given moment of time, and state *A* is determined by the same law by any of the system's previous states. This assumption implies a concept of reality, which defines this law, thus making this concept of causality ontological. My choice of the terms ‘classical causality’, rather than ‘causality’, used commonly, is due to the possibility, central to this article, of alternative, probabilistic, concepts of causality, applicable in QM, including in RWR interpretations, where classical causality does not apply. The term ‘determinism’ has been used to designate classical causality. I define ‘determinism’ as an epistemological category referring to the possibility of predicting the outcomes of classically causal processes ideally exactly. In classical mechanics, when dealing with individual or small systems, these concepts become equivalent. On the other hand, classical statistical mechanics or chaos theory are classically causal but not deterministic in view of the complexity of the systems considered, which limit us to probabilistic or statistical predictions concerning their behaviour. In these cases, however, the recourse to probabilities is merely practical: it is due to our insufficient knowledge of the behaviour of the systems considered in view of their mechanical complexity. By contrast, in QM, this recourse is unavoidable even in considering the most elemental quantum objects, such as elementary particles, which, besides, cannot be considered apart from their interaction with measuring instruments. This is because, as noted, the repetition of identically prepared quantum experiments in general leads to different outcomes, and unlike in classical physics, this difference cannot be diminished beyond the limit, defined by *h*, by improving the capacity of our measuring instruments. It follows that the probabilistic character of quantum predictions must hold in interpretations of QM or alternative theories of quantum phenomena (such as Bohmian mechanics) that are classically causal. Thus, while in classical physics, some theories are deterministic and others probabilistic or statistical, in quantum theory, the behaviour of all systems considered, individual, no matter how elementary, can only be treated probabilistically.

This fact as such does not exclude the classically causal nature of the ultimate reality responsible for quantum phenomena, thus making the recourse of probability only a practical matter similarly to classical physics when it used probability. In RWR interpretations, however, QM or QFT is not classically causal because the ultimate nature of reality responsible for quantum phenomena is assumed to be beyond a representation or conception. Classical causality would imply at least a partial conception of this reality. The recourse to probability becomes due to the absence of classical causality for all quantum systems considered, and hence, fundamental, irreducible rather than merely practical. It has nothing to do with, in Bohr's words, ‘statistical considerations as practical means of accounting for the properties of mechanical systems of great structural complexity’ [6] (v. 2, p. 34). Instead it is a consequence of the singular, unique nature and, correlatively, the essential indeterminacy or randomness of individual quantum phenomena.^6^ This randomness is not found in classical physics, because even when one must use probability there, at bottom one deals with individual processes that are classically causal and when it comes to the behaviour of its elemental individual constituents of classical systems, deterministic, thus making the recourse to probability merely practical. In classical physics, randomness does not ultimately exist or is assumed ultimately not to exist. Deterministic predictions are never possible in considering the behaviour of individual quantum systems, no matter how elementary, even though some of their effects on measuring instruments could be predicted exactly in certain circumstances.

One might, as noted above (note 6), distinguish between indeterminacy, as a more general category, and randomness, as a most radical form of indeterminacy, when a probability cannot be assigned to a possible event. Both indeterminacy and randomness only refer to possible future events and define our expectations concerning them. Once an event has occurred, it is determined. An indeterminate nature of events may either allow for assuming an underlying classically causal architecture (which may be temporal) of the physical reality responsible for this nature, whether this process is accessible to us or not, or disallow for making such an assumption. The first case defines indeterminacy in classical physics, such as classical statistical physics or chaos theory, or in considering the so-called algorithmic complexity in classical (Shannon) information theory, such as Kolmogorov complexity, which is not computable (the Turing machine will come to a halt), but if so, as finite, only for practical reasons, while assuming a determinate, if not trackable, structure of the process. The second is found in QM or QFT, and hence in quantum information theory, in RWR interpretations, where the complexity becomes infinite and hence not computable, in principle, fundamentally, as one cannot assume any determinate, even if not trackable, structure. This makes it difficult, if possible at all, to use the concept of the Turing machine, even the non-deterministic one, which is still finite and classically causal, in the present definition. The non-Kolmogorovian nature of quantum complexity is connected to the non-Kolmogorovian (non-additive) nature of quantum probability, but the subject would require a separate analysis. Quantum phenomena, however, only *contain* this irreducible randomness, rather than are strictly random, because they also contain correlations between multiple events (such as the EPR correlations, at stake in Bell's or Kochen-Specker theorems), which are strictly quantum and not found in classical phenomena. QM predicts these correlations, but at least in RWR interpretations, it does not explain them, any more than it explains how any single outcome of an observation comes about [1] (pp. 253–256).^7^

It is no surprise, then, that Bohr associates quantum phenomena only with events that have already occurred and not with possible future events, such as those predicted by QM on the basis of previous events, even if these predictions are made with probability equal to one, as is possible in certain circumstances. The reason is that a prediction for variable *Q* (for example, that related to a coordinate, *q*) cannot, in general, be assumed to be confirmable by a future measurement, in the way they can be in classical physics or relativity. If, say, after measuring *q* (which can always be done ideally exactly) at time *t*_0_, one predicts, by using QM, the value of *q* with a given probability, in certain cases even probability equal to one, at a future time *t*_1_ and then measured *q* time *t*_1_, one will obtain a predicted value, as is well confirmed by experiment. One can, however, always perform a complementary measurement, that of *p* (the momentum), which will leave any value predicted by using *Q* undetermined by the uncertainty relations, which in principle preclude associating a physical reality corresponding to a coordinate *q* when one measures *p* [1] (pp. 210–212). In classical physics, this difficulty does not arise because one can always define both variables simultaneously as referring to an independent reality. In any quantum experiment one deals with a system containing an object and an instrument that interferes in the ultimate reality responsible for the phenomenon observed and thus makes it different from the object considered, whether such it is assumed to be defined independently or only at the time of observation.

## 3. Causality as probability: quantum causality and the arrow of events

Bohr's final account of his interpretation in his 1958 article ‘Causality and Complementarity’ [6] (v. 3, pp. 1–7), also contains, for the first time in his work, a view of causality applicable in quantum physics, as opposed to classical causality, to which Bohr had referred by ‘causality’ previously. According to Bohr:

Although, of course, the classical description of the experimental arrangement and the irreversibility of the recording concerning the atomic objects ensure a sequence of cause and effect conforming with elementary demands of causality, the irrevocable abandonment of the ideal of determinism finds striking expression in the complementary relationships governing the unambiguous use of the fundamental concepts on whose unrestricted combination the classical physical description rests. [6] (v. 3, pp. 4–5).

There is no conflict with Bohr's previous appeals to the renunciation of ‘the classical ideal of causality’, because the view of causality invoked here is not classical [11] (p. 83) [6] (v. 2, p. 41). This renunciation is replaced here with ‘the irrevocable abandonment of the ideal of determinism’. This is the same ideal, because, as explained, in considering individual or simple classical systems determinism and classical causality are equivalent. On the other hand, Bohr only speaks of ‘ensur[ing] a sequence of cause and effect conforming with the elementary demands of causality’, due to the irreversibility of recording and thus the arrow of events, as correlative to complementarity, rather than defines the corresponding concept of causality. This is of some interest given that by this point Bohr's interpretation contained all the ingredients necessary to do so and that he previously spoke of complementarity as ‘a generalization of the classical ideal of causality’, without, however, ever explaining in what sense it is [11] (p. 83).

Quantum causality, as a form of probabilistic causality, is a concept that fits the bill on both counts, although it no longer contains the concept of cause, invoked by Bohr, only that of effect. This concept was introduced by this author previously (e.g. [1] pp. 207–218, [12] pp. 203–207), but it will be given here a new emphasis on the arrow of events. This concept originates in Heisenberg's thinking leading him to the discovery of QM. As he said, shortly before completing his paper containing this discovery: ‘What I really like in this scheme is that one can really reduce *all interactions* between atoms and the external world … to transition probabilities' (Heisenberg, Letter to Kronig, 5 June 1925; cited [13], v. 2, p. 242). By speaking of the ‘interactions between atoms and the external world’, this statement suggests that QM was only predicting, probabilistically, the effects of these interactions observed in instruments, without representing how these effects come about, a view that was adopted and developed by Bohr. This view, as explained, replaced measurement in the classical sense (of measuring pre-existing properties of quantum objects) with establishing, by using these instruments, quantum phenomena, which can be treated classically without measuring the pre-existing properties of quantum objects. Quantum causality defines the probabilistic nature of these transitions between observations or measurements. It connects a given measurement, A, to *possible* future measurements, also erasing the significance of any measurement preceding A, in predicting these future measurements, amplifying the role of the arrow of events.

I define first a more general concept of probabilistic causality. An actual event, A, that has happened allows one to predict which events *may* happen with one probability or another, but, in contrast to classical causality, without assuming that any of these events *will* happen (regardless of outside interference that can prevent predicted future events from happening in classical physics). Event A, at time *t*_0_ defines certain *possible*, but *only possible*, future events, such as X_1_ at time *t*_1_, and may exclude certain other possible events. In the case of quantum causality, A, as an event of observation and then measurement (of observed classical properties thus established) defines an ‘expectation-catalog’ for possible future events, within a given range, enabled by QM, cum Born's rule [9] (p. 154), and strictly precludes forming complementary expectation-catalogs. The temporal precedence of A vis-à-vis X_1_ or any X*_n_* at any future moment in time, *t_n_*, and the (local) arrow of events is inevitable. Each new event of measurement, A_new_, by virtue of interfering anew with the ultimate reality responsible for quantum phenomena, erases all connections, physical and informational, between any past event prior to A_new_ and possible future events defined by A_new_, which erasure ensures the arrow of events. All such predictions are quantum-non-local insofar as they are *predictions* at a distance, but they respect what may be called Einstein-*locality*, which prohibits any *action* at a distance (Einstein-*non-locality*), incompatible with relativity [1] (pp. 347–253). As explained above, one at *t_1_*, always has a free or sufficiently free choice to perform an alternative, in particular complementary measurement, and thus, by an alternative decision, establish an event, Y, and a reality different from the one predicted, X, even if this prediction was made with probability equal to one, as is possible in some cases. While this decision may not be entirely a matter of a free choice, the determination of a possible future thus obtained is local (technological, psychological or social), defined the agent's situation. Only the temporal precedence of A vis-à-vis either X or Y is definitive, conforming to the arrow of events.

Quantum causality, thus, pertains strictly to our interactions with the world by means of experimental technology: each event and the corresponding phenomenon are created by this interaction, rather than by a measurement of some pre-existing properties of the object or of the ultimate constitution of reality considered to be responsible for it, especially as defined by a classically causal process. Quantum causality does refer to our probabilistic knowledge concerning *possible* future events on the basis of our determinate knowledge obtained in previous *actual* events. The recourse to probability is, however, not due to insufficient knowledge concerning how quantum events come about. It is due to nature itself and our technological interactions with it.

The concept of quantum causality just outlined gives a meaning to Bohr's view of complementarity as a generalization of causality [6] (v. 2, p. 41). I cannot be certain that this is exactly what Bohr had in mind, but at the very least it is consistent with his overall argumentation. As against classical physics or relativity, implementing our decision concerning what we want to do will allow us to make only certain types of predictions and will exclude the possibility of *complementary* types of predictions. Complementarity generalizes causality because it defines which events can be probabilistically predicted by our decision concerning which experiment to perform. In addition to Bohr's 1958 anticipation, several concepts of probabilistic and quantum causality have been proposed in quantum information theory, without, however, relating them to complementarity, such as [14–16], discussed in [1] (pp. 207–219).^8^

Could one claim that the arrow of events only applies to the events of measurements due to these interactions and not to the ultimate reality pre-existing and responsible, in our interactions with it, for these events? Such claims have been made, but they and debates concerning them will be put aside here, because they are inapplicable in RWR interpretations. By placing this reality beyond representation or conception, these interpretations preclude all claims concerning this reality, including those associated with any concepts of event or temporality, whether defined by the arrow of events or time or not. In RWR interpretations, our claims only concern the temporal relations, from the present to the future, between events, as created by our interactions with nature and observed by us, which makes the arrow of events unavoidable. In these interpretations, apart from these interactions and, hence, us, nature has no events or temporality, any more than atemporality, however, either is conceived of.

I now turn to the idea of cause, to which, unlike Bohr's remark and the works just cited, the present concept of quantum causality does not appeal. As explained, quantum causality pertains strictly to the relationships between two events of observation, the initial event, A, and a possible future event, X, and not to what happened between these events, which is beyond all knowledge or even conceptions in RWR interpretations. This may invite one to see A as a cause of X, in contrast to classical physics. The *existence* of a classical cause or causes of an event, X, is commonly (albeit not without exceptions) assumed along with classical causality. It is, however, also commonly acknowledged that it may not be possible for us to claim the status of the cause for a given entity or set of entities preceding X, although the initial event, A, may be considered as embodying this cause or set of causes, such as those defined by gravity in considering the motion of a planet in the solar system. Either way, however, our observational intervention into the course of reality is neglected, as it can be for all practical purposes. Accordingly, the causes of events are independent of us and may not be known. By contrast, in quantum physics, our intervention into the course of reality in A defines a quantum phenomenon and as a result affects the future course of reality, which might appear to reinstate the idea of a cause. Is, then, A the cause of X, once X *has occurred*? The answer in the present view is no. First, one cannot be certain that X will happen, even within a probabilistically predicted range, because of A in any individual case. There is always a probability, however small, that it will not. Furthermore, as explained, even when our prediction concerning X can be made, at time *t_1_*, on the basis of the measurement, A, with probability equal to one, one can always make an alternative, such as complementary, measurement at time *t*_2_ which will disable the original prediction and define a different event Y at *t*_2_. One certainly cannot see A as the cause of Y, which, while an observation in its local environment, would no longer be related to A, as X would be. But, once Y happens, X is no longer possible. Hence, A cannot be a cause of X either.

What is always in place is the temporal precedence of A relative to either X or Y. Either event, X or Y, would respect this precedence, as neither can precede A, thus conforming and indeed defining the arrow of events, inherent in the concept of quantum causality. As noted, however, in strong RWR interpretations, the arrow of events, along with quantum causality, is only manifested classically in observable phenomena. What can be ascertained is that the ultimate nature of reality responsible for quantum phenomena is such that all our interactions with it by means of experimental technology entail the arrow of events, which quantum causality reflects. Hence, in strong RWR interpretations, the concept of the arrow of events cannot apply to the ultimate constitution of reality any more than those of change or motion. Nor, however, as discussed from the outset, could their reversals apply, which would imply that this constitution has no change or multiplicity but only permanence and oneness, as is sometimes suggested (e.g. [19]).^9^ In strong RWR interpretations, no concepts at all, including time or space, or ‘constitution’, could apply to this constitution.

In these interpretations, the equations of QM or QFT, such as Schrödinger's or Dirac's equation, are not equations of the motion of quantum objects. Moreover, in the present interpretation, quantum objects are only defined at the time of observations, which further precludes the application of any concept of motion (or any other concepts) to what happens between experiments. These equations only enable one (with the help of Born's rule) to form expectation-catalogs concerning the outcomes of future experiments, which implies the arrow of events. Accordingly, the formal time reversibility of these equations, sometimes used to argue against the arrow of time (the arrow of events is, again, rarely considered), has no physical significance. (Technically, to maintain the formal reversibility of the equations of QM and QFT with respect to *t* requires replacing variables by their complex conjugates, reflecting the mathematical structure of QM as different from classical physics or relativity.) Rigorously speaking, what is reversible in them is not *time*, but the value of the parameter *t* in these equations. *t* is connected to measurements, which respect the arrow of events, defined the ‘the irreversibility of recordings' invoked by Bohr, in the double sense—from a quantum phenomenon to the ultimate reality defining them and that of the later to an earlier phenomenon in any sequence, in which case the arrow of event, *in time*, or time, is manifested in our readings of the clocks [6] (v. 3, p. 5).

It might be added that symmetry considerations are crucial in considering and in fact requiring the arrow of events in QFT, as it is currently constituted. Using for the moment the standard language of time rather than events, time-reversal symmetry is prohibited insofar as quantum theory holds, in view of the CPT (charge, parity and time reversal) symmetry, a fundamental symmetry of physical laws under the simultaneous transformation of charge conjugation (C), parity transformation (P) and time reversal (T). CPT is the only combination of C, P and T observed to be exact symmetry of the ultimate constitution of nature, assuming that QFT holds. The CPT theorem says that any Lorentz invariant local quantum field theory with a Hermitian Hamiltonian must have CPT symmetry. This implies that every violation of the combined symmetry of two of its components, say, CP, must have a corresponding violation of the third, such as T.^10^ We know, however, that CP is experimentally violated, because P is violated in weak interactions, which was an unexpected 1956 discovery, for which T.-D. Lee and C. N. Yang (who predicted it), and C.-S. Wu (who experimentally confirmed it), won the 1957 Nobel Prize. It follows that, even if one puts aside the irreducible role of observation, which guarantee the arrow of events in quantum physics, this arrow would still be in place by the CPT symmetry, applied to the parameter *t* in the equations of QFT, a parameter necessary to predict events that might happen at particular moments in time, corresponding to the readings of the clocks used.

In RWR interpretations, the arrow of events is only manifested classically in observable phenomena, as an effect of our interactions with matter, which is ultimately beyond all conception, by means of experimental technology, beginning with our bodies and brains. The latter are, however, not sufficient to observe quantum phenomena, which require additional experimental instruments, from silver bromide plates to the present-day particle accelerators, to be observed, by essential interfering with physical reality. The necessary use of such instruments is responsible for the difference between the ultimate constitution of this reality or quantum objects and quantum phenomena, beyond that defined by our bodies and brains. The arrow of events is unavoidable in this view, according to which physics, *all physics*, is not about how nature ultimately is but about our interactions with nature by means of experimental technology beginning with our bodies, and our thinking. These interactions entail the arrow of events and the corresponding temporality (the arrow of time), manifested in our *conscious* thought. It is just that in quantum physics, or already in relativity, our bodies are never sufficient to observe quantum phenomena, which requires special instruments. While we do use observational instruments, such as telescopes, in classical physics, our bodies may be sufficient in some cases, as, paradigmatically, in observing the motion of planets around the Sun. Our bodies are, however, never sufficient in quantum physics or relativity, although in relativity we can still treat the behaviour of objects considered as independent of the interference of the instruments, except our bodies.

In this view, then, time belongs to human thought, and especially as associated with the arrow of events in time, only to conscious thought, from which the time used in physics, the time defined by clocks, is constructed by idealization. It is not clear that conscious temporality and its arrow, as, for example, conceptualized by E. Husserl, as a linear sequence of present moments in time or *Nows*, A-B-C (where *Now* B is the retention of *Now* A and *Now* C is the protention of Now B) would be obeyed by the unconscious, which, at the very least, scrambles it (e.g. [22, p. 67]). It is also not clear that this temporality would be obeyed by nature, at the ultimate level of its constitution, at least of that part of this constitution that is responsible for quantum phenomena, but I would argue in general. Our interactions with the world in physics and beyond depend on consciousness and its arrow of events *in time*, even if nature or our own unconscious, does not obey this arrow. In RWR interpretations no concept of temporality or that of event (and hence that of the arrow of time or events or, conversely, the absence of any such arrow) can be ascribed to nature itself.^11^

This need not mean that our *interactions* with nature by means of experimental technology and human thought, including the mathematics of quantum theory, should be seen in *subjectivist* terms, even as concerns our *experience* resulting these interactions. It would be difficult to see as subjective either nature or technology, even if only that of our bodies, let alone that used in quantum experiments, especially now. This is not impossible, say, along the lines of Plato's or, in modern times, Bishop Berkeley's argument, which denies the existence of anything beyond thought, and hence both material nature and technological devices built of material parts. Plato and Berkeley only permit the technology of thought, such as mathematics, on which Plato indeed modelled his philosophy. Our experience arising in the interactions with nature in quantum physics does contain subjective elements, such as those involved in our assignments of probabilities, say, along B. de Finetti's lines [24], especially if these probabilities are, as in RWR interpretations, not due to a lack of knowledge of how quantum events come about. If they are, then the subjective nature of these assignments could, at least in principle, be overcome by objective knowledge of the reality behind quantum events, in the way it is in principle possible, or is assumed to be possible, in classical statistical physics. ‘Personal’ might be a better concept insofar as these assignments are shaped by things in the world and human interactions between agents. Things are rarely, if ever, completely subjective, permitting that such exterior factors are interiorized at the time of an assignment of probabilities of future events. At the same time, any quantum experiment that has been performed gives a definitive and unambiguously communicable (classical) outcome. An agent cannot control it but can only predict it probabilistically by means of QM (cum Born's rule). One might be able to decide which observation or measurement to perform, for example, one or the other complementary observation, but one cannot control the specific outcome of it as concerns which value one obtains. This makes measurement objective in this double sense—the lack of control of an outcome and the possibility of an unambiguous communication of an outcome—but in the present view, only in this sense, rather than *objectively* attributing anything to nature itself beyond its existence and, as part of it, human existence. An observation in quantum physics is a unique act of creation, performed by a particular agent or several agents, and as such it has personal aspects, including those shaping our decision leading to this act. Once the measurement is performed, however, the outcome is fixed as a permanent record, classical in character, and hence it can and, in science does, function objectively. It may be unknown to others, but that is not the same as being subjective, because it can become known to others. It is also true that, as any record, it must still be experienced by somebody to be meaningful. Still, while an *act* of observation or measurement is personal (if sometimes determined collectively), its *outcome* is not. It can be experienced differently by different agents, and as such it has personal aspects.^12^

On the other hand, the future is never objective, because it is has not yet happened, which is one of the defining aspects of the arrow of events. QM or QFT allows us to estimate certain possible future events with one probability or another, without, in RWR interpretations, allowing us to know or even to conceive of why these estimates work, just as quantum experiments do not allow us to know or even to conceive of how what is observed in them happens. In other words, we do not know and possibly cannot conceive of how quantum physics ultimately works. But, luckily for us, it does.

## 4. Thinking as singularity: quantum-like theories in RWR interpretations

This section considers the implications of the preceding argument for the mathematical modelling known as quantum-like, Q-L, modelling, versus classical-like C-L, modelling, based in the mathematics adopted from classical physics, and Q-L theories beyond physics—in psychology, cognitive science, decision sciences, and so forth, in short, using the term more common in French, human sciences. Q-L theorizing and modelling is by now an extensive and rapidly expanding field. I assume that, as in quantum theory, that a Q-L model refers to using the quantum-mechanical or analogous formalism, and a Q-L theory to the overall conceptual architecture, including the model, necessary to account for the phenomena considered. Although our thinking is commonly assumed by Q-L theories (and will be assumed here) to be due to the neurological workings of the brain, it is not necessary to assume (and will not be assumed here) that the aspects of human thinking treated by these theories arise from the quantum physics of the brain. Q-L theories may apply even if the physics of the brain is classical. There are hypothetical theories that consider consciousness or thinking to be an effect of the quantum physics of the brain, such as, prominently, those by R. Penrose, beginning with [27], and his followers. Such theories will, however, be put aside here. How the physics of the brain makes thinking or consciousness, as we experience it, remains an unanswered question, sometimes referred to as ‘the hard problem of consciousness’, the problem of explaining the possibility of qualia (qualitative phenomenal properties, such as experiment of a colour, say, green, versus its physical nature, defined by the frequency of the corresponding light) [28]. The appeal to consciousness appears to be due to the fact that our manifested inner experience is that of consciousness, and not of the unconscious, usually *inferred* by theoretical means from our conscious thinking. While assumed to be responsible for mental processes, the brain will be treated here as, *physically*, a black box, relating the informational input and output, encountered from either the outside or the inside of a human subject. In the present view, rather than merely unknown, the workings of this black box are beyond conception, in parallel with strong RWR interpretations of QM, where the black box is that between phenomena registered in observational instruments in quantum experiments.

I argue more generally that what is shared by quantum theory, QM (to which I shall continue to restrict myself) or QFT, and Q-L theories is their Q-L ontological and epistemological architecture, especially the irreducible interference of observation, complementarity, probabilistic causality, and the arrow of events, all interpreted in RWR terms [29,30]. It follows and, in fact, is assumed first that in Q-L theories, an observation is a creation of phenomena by means of the interactions between one or another agency with the ultimate reality considered (such as certain strata of matter or thought, especially the unconscious) rather than observing or measuring the pre-existing or even contemporaneous properties of this reality. In strong RWR interpretations, the ultimate constitution of the reality responsible for thinking cannot be *thought* (even if one leaves aside the role of the brain), any more than that of the material reality responsible for quantum phenomena could be in strong RWR interpretations. The first reality is, however, unique to each of us, while the second is shared by us.

To recapitulate the key relevant points of the preceding analysis, in quantum physics our decisions concerning which experiments we perform, *always*, *in all possible cases*, essentially affect, by interfering with reality by means of observational instruments, what may or, specifically, by complementarity, may not happen with one probability or another. These decisions define the course of reality, in contrast to classical physics or relativity, where our experiments, in principle, follow what would happen in any event. In quantum physics, one *decides*, say, at time *t*_1_, which experiment to perform, either the one concerning the values of the position or that concerning the values of the momentum. This decision then enables one, by using QM, to estimate the probability of finding either the position or the momentum of the object within a certain range at a subsequent moment in time, *t_2_*. At the same time, performing either measurement in principle precludes predicting such a probability for the complementary variable. If one performs two such measurements in sequence, the outcomes will be different depending on the order in which they are performed, because each creates a singular, unique measurement situation. As, however, explained in §2, doing so requires two different experimental arrangements, and two different quantum objects. This fact, underlain by the irreducible role of observational instruments and the irreducible individuality of each event, is a manifestation of complementarity pertaining to both the initial measurements used and the two alternative sequences considered, and hence quantum-mechanical predictions concerning the outcome of possible experiments.

By contrast, in Q-L phenomena, as in cognitive psychology and decision science, say, in experiments based on asking a given question or set of questions, the *object under investigation* is a *human subject*, the system comprised by consciousness and the unconscious of this subject, a C-UC system. We keep in mind that one is dealing with mental rather than physical reality, bracketed even though a physical reality, that of our brains, is responsible for this mental reality. In quantum physics, which concerns independent (and possibly RWR) physical reality, one never deals with any decision on the part of *the objects under investigation*, because these objects are not human subjects. Each human subject, defined by a C-UC system makes a decision in response of the question asked. This decision, as communicated to the agent, is manifested to the subject consciousness, although the unconscious of the subject may affect or even define it, thus bringing in the C-UC system. In this case, one can see consciousness as performing a kind of ‘measurement’ on the unconscious as unknown reality, akin to that responsible for quantum phenomena (e.g. [29–31]). The difference between quantum and Q-L situation is still in place, even though in quantum physics one investigates the system consisting of the object and the instrument, neither of which is a human subject. There have been arguments that consider quantum objects as differently ‘responding’ to different experimental setups, or even possessing something akin to consciousness or memory. I am reluctant to ascribe any human attributes to inanimate nature. It is more reasonable to see the situation in terms of different *types* of interactions between quantum objects (the latter, moreover, being only defined at the time of observation in the present interpretation) and observational instruments. I add types, because in the present view any actual interaction is different, insofar as it, in general, leads to different outcomes, in quantum physics.^13^

To illustrate the difference between quantum and quantum-like phenomena just sketched, I shall consider a claim by Z. Wang and G. Busemeyer in their discussion of complementarity in psychology via the experiment, paradigmatic in Q-L approaches [33]. Two questions are asked, ‘Do you generally think President Clinton is honest and trustworthy?’ (which, if asked by itself, tends to elicit the negative answer) and ‘Do you generally think Vice President Gore is honest and trustworthy?’ (which, if asked by itself, tends to elicit the positive answer) in two sets of trials by reversing their orders. These sets exhibit two statistically different outcomes. I have discussed the case in detail in [30], and I shall only reprise those aspects of it not addressed in [30], such as the nature of causality in this and related experiments.

According to Wang and Busemeyer: ‘Once we obtain a measurement on say, Clinton, that decision can create a definite position for Clinton, but then the opinion regarding Gore must be uncertain’ [33] (p. 2). This is not entirely accurate, or at least not sufficiently qualified. The opinion of the subject regarding Gore is uncertain for an outside agent, who could, in any event, never be certain what the subject actually thinks, even if the subject provides as much information as possible concerning this thinking. In fact, subjects cannot be entirely certain, in their consciousness, about their thinking either because of the unconscious. This is a fundamental defining feature of human interactions (akin to that of the ultimate reality in quantum physics, which can never be known or even conceivable to us) and it affects the Clinton–Gore experiment as well. The opinion concerning Gore may be certain for the subject, alongside that concerning Clinton. This makes the situation different from quantum physics, where this type of uncertainty is indeed in place because of the uncertainty relations and complementarity. In quantum physics, one, in considering any complementary situation, only deals with the decisions of agents concerning which measurement to perform as the initial measurement, say, at time *t*_0_, which also determines what kind of predictions one can make concerning future measurements, which can test our predictions, say, for one at time *t*_1_. As explained, however, measuring, as one can always decide to do, the complementary variable at *t*_1_ will irrevocably preclude verifying the initial prediction. Our decision, including our (sufficiently) free choice, which observation to make is a crucial part of Bohr's complementarity and is correlative to quantum causality and the arrow of events. It is difficult and arguably impossible strictly to instantiate this conceptual structure in psychology or decision making.

Can, nevertheless, these aspects of Bohr's concept, and with them, a form of probabilistic causality and the arrow of events, rather than only the mutually exclusive nature of certain phenomena, be transferred to cognitive psychology and decision science, this bringing both *closer* together? Yes, they can, even if only partially. It is not, I would argue, possible to do so entirely. This can be done by adjusting, vis-à-vis Wang and Busemeyer's claim above, the view of what happens once one obtains an answer concerning either Clinton or Gore, as the first answer in the sequence considers. Before I explain how, I add a few qualifications, which reflect the limits of this transfer, again, only partial.

First, in the Clinton–Gore experiment or most Q-L experiments, the statistics of the outcomes are at least in part defined by *classically causal* relationships between the two answers, ‘measurements’, in a given sequence, for well-established psychological reasons. One can expect these answers and their (rough) statistics in advance because of these classically causal relationships. In the case of quantum phenomena, at least in RWR interpretations, there are no classically causal relationships *in any situation*, and if there are, they are much more difficult to establish and are certainly debated. There is no such debate concerning the classically causal psychological relationships just indicated, even if only *in some situations*. This may be one of the reasons that, while Q-L models deal with non-additive, non-Kolmogorovian, probabilities, they are rarely the same as QM mathematically, and they are multiple, as opposed to the single theory, QM or, in high-energy regimes, QFT, predicting all quantum phenomena. There is no Planck's constant, *h*, either, which is responsible for the mathematics of QM *in all of its aspects*, and which, as noted, reflects certain fundamental aspects of nature, assuming that QFT holds all the way down to the Planck scale. In quantum physics, at least in RWR interpretations, once one measures one complementary variable, say, the position, no expectations at all are experimentally possible as concerns a future value of the other at any future point. In fact, this is true even if one assumes classical causality, as in Bohmian mechanics. In other words, when one is asked the second question, concerning the momentum, in any given sequences, the *information* carried over from the preceding question, concerning the position, *does not and, in principle cannot,* influence the response to this new question, or the future predictions from this point on. According to Wang and Busemeyer: ‘When one is asked a subsequent question, information carried over from the preceding question provides a context for the construction of the second and influences the subsequent response’ [33] (p. 2). That may often be true in psychology and human decision making, but is never the case in quantum physics, beginning, again, with the fact that quantum objects do not possess information. No information carried from an earlier position measurement (answering the question about the position of the object) has any relevance for any subsequent momentum measurement (asking the question about the momentum of the object). This fact renders this earlier information meaningless as concerns future predictions. Each quantum measurement is unique, and non-commutativity of QM reflects this uniqueness, including in the case of two *reversed* complementary measurements, which give us different outcomes, in contrast to classical physics. There these outcomes are the same, because, neglecting, as one can, the interference of observational instruments, both variables can be determined jointly at any moment in time. Even if one measures only one, the other is definable, thus reflecting the fact that one decides to know one or the other aspect of *the same reality*, which is not complementarity in Bohr's sense. The future course of reality is classically causally predetermined as well. There is no uncertainty relation or complementarity *in Bohr's sense*. By contrast, in deciding to establish one or the other complementarity phenomenon in quantum physics one defines *a new, singular or unique, reality* and a new, unique, future course of reality.

What is, however, akin to quantum physics in Q-L human situations is the essential individuality or singularity, uniqueness, of human thinking of each person and of each of its instances [30,34].^14^ This singularity is a key aspect of the Q-L situation, which is, I would argue, missed or underappreciated by Wang and Busemeyer's discussion of complementarity in either quantum physics or psychology. It is, however, this individuality that allows us to bring quantum physics and Q-L theories of human thinking closer, while still respecting the differences between them, as outlined above. The parallel is *partial*, but it is meaningful and is correlated with the need to use some form of Q-L formalism (it may not be the same as in QM) in assessing the statistics of responses in the Clinton–Gore or other Q-L experiments.

First, asking the subject under investigation *any question* by an agent may be seen as quantum-like interference into the unknown and ultimately unknowable reality of the subject's thought which elicits a conscious response, observed by the agent, akin to the way observation works in quantum experiment. Accordingly, as in the Clinton–Gore scenario, this interference defines a future thinking of the subject, strictly conforming to the arrow of events, just as an observation would do in quantum physics. Thus, if one asks the question *just about Clinton*, the subject thinking becomes oriented in a particular way, which will shape the subject answer, in accordance with this orientation, concerning the subject's attitude toward Gore, if such a question is asked next. It is not that the subject opinion concerning Gore *necessarily* becomes uncertain, as Wang and Busemeyer contend. This opinion might just not be there at all, or if it is there, either in consciousness or the unconscious, it *might* become reoriented as a result on the question about Clinton. (This is not certain either, but the possibility is statistically relevant.) The same *type* of structure is in place if the first question is just about Gore, although the specific outcome may be different, because we deal with an alternative situation of observation, mutually exclusive with the first. These two situations are complementary, now more in accord with Bohr's concept, because one deals with two mutually exclusive possible courses of reality, each of which is complete by itself, rather than merely representing two different parts of the same reality. In other words, while these two questions or answers, concerning trusting Clinton or Gore, are not complementary by themselves, the two alternative sequences in question may be seen as complementary, leading to the statistics obtained. By contrast, in quantum physics, two such sequences are complementary because the measurements of two variables involved, such as that of the position and that of the momentum, are always complementary in the first place, correlatively to the uncertainty relations, for which there is no real analogue in Q-L theories, which have no *h*. Non-commutativity alone is not enough for the uncertainty relations or complementarity.

By the same token, just as in quantum physics, the arrow of events is strictly respected in the Clinton–Gore experiment, because one only deals with the future course of reality defined by the first question and answer to it. It is crucial that, just as it is in the case of complementarity in quantum physics, that each alternative reflects the only course of reality there is, rather than a half of the same reality. Of course, in each alternative situation, the agent prediction concerning the second question in the order will be different, depending on their order, and, it appears, will obey Q-L non-additive probability and statistics, as do most such Q-L experiments beginning with A. Tversky and D. Kahneman pioneering work [35,36]. The observed outcomes will be statistical, because the reversal will in general concern different subjects. But then, so they are in quantum physics, where, as explained, complementarity experiments, either individual or sequential, are performed on different objects as well. In both cases, one deals not with classical but probabilistic causality, correlative to the arrow of events.

The main difference remains the stratification of the situation in psychology or decision making not found in quantum physics. In quantum physics, alternative (complementary) future courses of reality are determined by the alternative decisions, allowing for a sufficiently free choice to an agent, in interaction, by means of experimental technology with physical reality. Any quantum data can only be created by this technological interaction and not otherwise. This decision always defines an alternative, mutually exclusive, reality and the future course of reality, thus combining the mutual exclusivity of the phenomena considered and the role of an agent's decision. In human Q-L phenomena, an object under investigation, as in the Clinton–Gore experiment, is a human subject, and one deals with a decision by this subject *elicited* by the question coming from an agent. This question, too, can be defined by a decision of those conducting the experiment, but this does not change my main point that the observation that defines the corresponding course of reality is made from within the thinking, including possibly the unconscious, of the subject. This decision defines, from within the subject, the ultimate reality considered, analogously to the decision of the human agent performing a quantum experiment.

This analogy is limited, however, because though in Q-L psychological experiments, this decision may be determined by the question of the agent. The subject does not decide which question to ask but only which answer to give. In quantum physics, a quantum object, by interacting with the instrument in one set-up or another, determined by the agent decision (as a question one asks from nature), produces an outcome, defining the future course of reality, including in a complementary situation as mutually exclusive with the complementary alternative. But quantum objects or nature do not make decisions or answer questions, except speaking metaphorically. Nature only allows us, as human subjects, to obtain answers to questions we pose by using very specific measuring instruments, required by quantum experiments. (There is no need for such instruments in psychological experiments.) Human agents ask (or answer) questions or make decisions, defining, with the help of nature, the state and the future course of reality. Quantum objects do not ask questions or make decisions, and in the present view, by the Dirac postulate, they are only defined by us at the time of observation. It is difficult (although not entirely impossible, say, along Bishop Berkeley lines) to assume that human subjects, as objects under investigation in Q-L experiments, are only defined at the time when questions are asked, even though their responses are so defined. Human subjects do change their thinking, and this change can be affected from the outside. It is, however, still generally assumed in psychological experiments that human subjects, as thinking beings, exist independently, each defining the reality of their thought, even if the ultimate nature of each such reality may be RWR, just is the ultimate nature of the (physical) reality responsible for quantum phenomena. This reality is, however, shared by us, even if its effects are experienced differently by us.

The ‘reduction’ of human reality, sometimes known as the Galilean reduction (that of physics as only dealing with nature versus as in Aristotle both nature and thought), made modern physics, from Galileo and Newton to relativity and quantum mechanics, and beyond, the mathematical-experimental science of nature, with mathematics coming first in this conjunction. This is not only or primarily because of the numerical role measurement versus qualitative observation in Aristotle (important as it may be) but also because the capacity to use the mathematical formalism to represent, classical causally, the physical reality considered and, by using this representation, to predict the outcome of experiments. In the case of individual or simple systems, this can be done, ideally exactly, deterministically. Probability eventually became used, but as discussed above, it was underlain by classical causality and hence was merely a practical tool. In quantum theory, this representation and classical causality became difficult to maintain, because determinism was no longer possible even in considering the simplest individual quantum systems, such as those associated with elementary particles. Following QM, such a representation or even a concept of how quantum phenomena come about became in principle precluded in RWR interpretations. This made probability irreducible, defined by quantum causality as probabilistic causality, and correlatively the arrow of events. QM, nevertheless, found a way to use very abstract mathematics, such as that of Hilbert spaces over C, to enable probabilistic predictions strictly in accord with what is experimentally observed, thus still enabling QM and then QFT to remain mathematical-experimental sciences, with mathematics playing an even greater role in them.

It is, however, rarely, if ever, noticed that the Galilean reduction is twofold, in fact threefold, because the mathematical-experimental nature of modern physics is already predicated on the double reduction. The first is defined by only dealing strictly with physical (rather than also mental) reality; and the second is defined by a mathematical idealization of natural processes, which requires disregarding those aspects of nature that cannot be mathematically idealized. There is, however, a third reduction. It is based on disregarding the multiple realities of each individual subject and considering only the single physical reality of nature, assumed to be (ideally) representable in the same way for all subjects considering it, at least in doing physics.^15^ This reduction is much more difficult, and perhaps ultimately impossible, except in *relatively* simple cases, in psychology, cognitive sciences and decision science, or elsewhere in human science, such as in economics, in short in any field expressly dealing with human thinking.

One can consider this situation in information-theoretical terms. The data considered in physics are a form of information, which can be treated mathematically as Shannon information (a collection of bits). Shannon information, considered in information theory, is based on disregarding the semantic content of information. This content is essential to our thinking and language in general (this also connects this content to the hard problem, as considered in detail in [29,30]), but is, I argue, only mathematizable to a very limited degree. The mathematical conceptualization and reduction of information in information theory was, however, a remarkable and important development with numerous theoretical implications and practical applications. It may be seen as an extension of the Galilean reduction in all three aspects of it just discussed. The (mathematical) information theory may be classical, which deals with information processing by means of classical physical systems, or quantum, which deals with information processing by means of quantum systems. The latter type of information cannot be created by using classical systems and, hence, obeys different principles of processing, and in this sense, it may be seen as quantum information, but, in the present view, *only in this sense*. This is because this information itself, qua information, is classical, Shannon information, observed by human agents in observational instruments used in quantum physics. There is no other information, but there are different ways to create and to process classical information, specifically classical versus quantum. In the latter cases, this classical information is obtained, by (necessarily) using quantum observational technology, in the experience of human subjects and is communicated, by means of language (involving but not limited to mathematical or technical terminology) to other human subjects. This communication is more easily made unambiguous and thus conforms to the requirement of modern, post-Galilean, physics as a mathematical-experimental science of natural phenomena, which are experienced by human agents, helped by observational technology. The latter is, again, auxiliary in classical physics and relativity, but constitutive in quantum physics, which leads to at least the possibility of RWR interpretations, including in dealing with quantum information and its complexity.

As discussed earlier, in considering the complexity arising to the indeterminacy in classical physics, such as classical statistical physics or chaos theory, or the algorithmic complexity in classical (Shannon) information theory, one encounters at most the Kolmogorov complexity. This complexity is not computable (the Turing machine will come to a halt), but if so, as finite, only for practical, epistemological reasons. In quantum information theory, and thus in QM or QFT in RWR interpretations, this complexity is not computable, in principle, fundamentally, rather than only in practice by virtue of the nature of the reality ultimately responsible for quantum phenomena. This makes it difficult, if possible at all, to use the concept of the Turing machine, even the non-deterministic one, which, while it uses probability, is still finite and classically causal. This radical (non-Kolmogorovian) informational complexity and epistemology are shared by quantum theory and Q-L theories (including those using Q-L mathematical models) in RWR interpretations.^16^

Quantum theory, however, can still use its mathematics to predict all the information found in quantum phenomena, information classical (Shannon) in character, but created by quantum means, defined by the complexity beyond the Kolmogorov complexity of classical (Shannon) information theory. While still dealing, as opposed to multiple human realities, with the single ultimate physical reality, including as responsible for quantum phenomena, QM or QFT further complicates this situation, correlatively to complementarity. In conjunction with quantum causality and the arrow of events, complementarity defines the state and the future course of reality in two or more alternative ways, by the decision of a human agent and, thus, this agent's individual inner reality.^17^ Still, as Bohr emphasized, the situation is in accord with the demands of unambiguous communication and, in this sense, objectivity, defining the project of modern science, from Galileo on. The difference, in RWR interpretations, is epistemological, insofar as we deal with RWR and, as a result, the infinite, beyond-thought, complexity of the way in which the information contained in quantum phenomena, unique each time, is created.

Linguistic communications may also be unambiguous or at least sufficiently unambiguous, as in describing experimental arrangements in physics or in presenting scientific concepts, including mathematical ones (which still cannot entirely avoid ordinary language), or philosophical concepts. As noted, Aristotle's physics was a great example of using language in this way. Galileo's dialogues would be another such example. In general, however, statements in, and concepts of, ordinary language leave a greater space for ambiguity and uncertainty as concerns the limits of their applicability (and even in science this ambiguity cannot always be completely avoided), as noted by Heisenberg [7] (p. 92). Hence, while human sciences are still sciences, the information they consider is much more difficult to contain by Shannon information, which complicates the use of mathematical models, either C-L or Q-L, there, especially in dealing with cognition or *thinking* (as a more general category adopted in this article). This is not only because the information supplied by human subjects is difficult to mathematize in an informational-theoretical way, but also that this mathematization cannot be certain to the degree (for all practical purposes, a full degree) it can be in modern physics. It is difficult to expect a comparable effectiveness of mathematics, sometimes puzzling, as famously to E. Wigner, even in natural sciences [37], in human sciences, where the Galilean reduction may only be partially possible.

That, as noted from the outset, need not mean that such theories are impossible or bound to have only little effectiveness. The question is instead that of the conditions and limits of their effectiveness, including in considering C-L versus Q-L theories. Given the role of complementarity, probabilistic causality and the arrow of events, especially if RWR interpretations apply, one might expect Q-L theories to be more effective than C-L ones. Even in simple cases, such as that of the Clinton–Gore experiment, our thinking appears to be able to produce decisions that C-L theories may not be able to handle, even though the issue does not appear to be entirely settled. As Wang and Busemeyer observe:

Unfortunately, it is true that compared to quantum physics, which provides rigorous and precise predictions about physical phenomena, psychological theories involve many more random variables that are hardly controlled, resulting in lower precision in prediction. To be fair, this is a general challenge that could be raised for any theories in the behavioural and social sciences. However, through rigorous model comparison, empirical studies have shown that quantum models provide an elegant new way to specify general and vague verbal theories in psychology, and better explain and predict many phenomena puzzling to classical models, leading to highly testable models (e.g. [38–40]). [33] (p. 4)

A long list of works, some proceeding more along RWR lines, supporting this view can be added (e.g. [41–43]). What I have argued in this article is that, rather than only a greater effectiveness of Q-L mathematical models, there may be deeper epistemological reasons, defined by the combination of features, such as the irreducible role of observation, complementarity, probabilistic causality and the arrow of events, that justify and may require the use of Q-L *theories*, underlying this mathematics, just as such features require the mathematics of quantum theory in physics. However, the richness and complexity of human thinking, limits the range and effectiveness of Q-L theories, thus far used in *relatively* simple cases. This richness and complexity is captured by T. S. Eliot's lines:

In a minute there is time
For decisions and revisions which a minute will reverse.
(‘The Love Song of J. Alfred Prufrock’, 1915, ll. 47–48)

We are far beyond Shannon information here, literally infinitely far, when it comes to informational complexity, the complexity of what is responsible for this information, if one adopts, as I do here, RWR interpretation of this situation and thus how such decisions and revisions come about. Eliot's ‘Do I dare?’ is also a decision question, and he repeats it ‘Do I dare or do I not dare?’ in the poem several times, even asking in the lines immediately preceding the one here cited: ‘Do I dare/Disturb the Universe’, which is what we always do in quantum experiments (ll. 45–46). These questions also represent the nature of Eliot's creative process in writing the poem. For the moment, it is clear that there are far too many qualia (qualitative phenomenal properties), the existence of which are the source of ‘the hard problem’, that one can experience in a minute, during which ‘there is time for decisions and revisions that a minute will reverse’, to be predicted by any mathematical model. This is what literature, such as Eliot's poem (only one of many examples) appears to tell us [30]. Even a second can reverse such decisions and revisions, while at the same time making each unique and unrepeatable. Eliot, including in this poem, undoubtedly made and reversed his decisions many times in choosing many words, reflecting many qualia. When one deals with the likes of Marcel Proust's *In Search of the Lost Time* or James Joyce's *Finnegans Wake*, the play of decisions and revisions becomes immense [31].

Intriguingly, C. Shannon considered the linguistic play of *Finnegans Wake* in illustrating his theory of Shannon information [44]. As I have argued in [30,31], without considering Shannon's analysis of Joyce, but my point is valid independently. Shannon information is not irrelevant, but it relates to a very small and often negligible part of the information contained in most novel's sentences, to many of which event the category of a sentence does not apply, or even words, on which Shannon focused in his comments. There are indisputably aspects of Joyce's wordplay that Shannon information theory can help one to analyse. To understand? That is a different matter. I would argue that Shannon information theory offers little for our experience of words, sentences and so forth in Joyce's novel (or literature in general), as defined by qualia.^18^ For example, a linguistic fusion of chaos and cosmos into ‘chaosmos’, one of the novel's famous coinages, has a Shannon-informational aspect to it, but this aspect tells us little about what chaosmos means in Joyce. For example, does it refer to something like a classical-like ultimate reality, say, that of the chaos-theoretical type, or quantum-like, RWR-type ultimate reality? As I have argued from the outset, these are two very different ways of mixing chaos and order, and these not the only such ways, especially if one moves beyond scientific ways of this mixing, which Joyce surely contemplated and staged in his text. The novel does say, undoubtedly referring to its own project, making the ‘I’ in the sentence that of Joyce: ‘I am working out a quantum theory about it for it is really most tantumising [a play on tantalizing] state of affairs' [46] (p. 149). At the surface level, the shift from ‘tantalizing’ to ‘tantumising’ may be seen in terms of Shannon information, but only at the surface level, for it still involves a play of qualia. But, apart from the fact that this sentence alone does not tell us how the novel configures thus quantum theory, the ‘quantum theory’ constructed by the novel extends beyond physics is beyond physics, is, as it were, quantum-like, which, again, can be interpreted differently. One would need to read and interpret the whole novel to address these questions, which are unlikely to be definitively settled beyond individual interpretations, and these, too, can change.

Joyce's or other literary compositions may and often do contain epistemologically quantum-like features, for example, a kind of entangled compositional structure, in which case, in Schrödinger's words, ‘best possible knowledge of the whole does not necessarily include the [best possible knowledge] of its parts [9], p. 166), the situation instructively considered in [47,48]. Still, such features, which are conceptually formal, would account for only a small part of the semantic, qualia-defined, content of compositional arrangements in literature or other art, including musical *compositions*, ultimately dominated by their qualia content. It would be even more difficult to handle mathematically how Joyce's own innumerable decisions and revisions (quite literally speaking) in writing the novel, or (less complex as these may be) the decisions concerning one or another reading of Joyce, come about. In the present view, these processes are more likely to be Q-L than C-L epistemologically, more likely to obey the (infinite) complexity beyond the Kolmogorov complexity [29,30,34]. It is also the present view, however, that this complexity cannot be handled mathematically in the way that of quantum phenomena can be, luckily for quantum physics. Still, we can write and read literature and think about it, by making decisions and revisions which a minute can reverse. In addition, artistic compositions have and are designed to have an aesthetic appeal to our feelings, an *affect*. All art, every work of art, is defined by this combination of a composition and an affect of this composition when experienced, and this experience is always connected and usually defined by qualia [49] (pp. 191–194). This combination is not absent elsewhere, even in mathematics and science, but it *defines* literature or art. As concerns the content and the workings of thought, however, literature may not only be an extreme example of these qualia-oriented workings but is also a model for them elsewhere. This model would impose formidable and, arguably, unsurmountable limits upon the mathematical modelling of these workings, even the Q-L one, which may be our best mathematical modelling of thought. This, again, does not mean that such models are inapplicable or, within their proper scope, ineffective. They are, and we need to continue to develop them.

But then, we cannot be sure either that quantum theory will ultimately be able to capture the richness of the ultimate reality of nature. As, in the strong RWR interpretations assumed here, a reality beyond the reach of thought, the ultimate constitution of physical reality may be a more complex reality than any we can imagine, literally speaking. The same may be true when it comes to the ultimate working of thought or (the hard problem) how the brain creates thought. The assumption that a reality is beyond thought allows for the possibility of this reality to be literally *unimaginably* rich or, in any event, different from anything we can imagine. It is likely that, limited by our evolutionary biological and neurological nature, we can imagine little of how the ultimate constitution of nature ‘is’, to the degree the word ‘is’ applies to this constitution and it would not if strong RWR interpretations hold. Bohr is reported to have replied, after the rise of quantum physics but before QM was discovered, to H. Høffding's question ‘Where can the photon be said to be?’ with ‘To be, to be, what does it mean to be?’ (cited in [50], p. 131). It may not be possible to speak about being of a photon, or even of a photon, apart from an observation associated with something that we call a photon. Still, in addition to giving us mathematical means to deal with phenomena in the world around us and thus to continue the mathematical-experimental project of modern science, quantum theory and quantum-like theories offer us new ways of thinking about phenomena in considering which one can only use mathematics in a limited way or cannot use it at all.

## Data Availability

This article has no additional data.

## Footnotes

1 This definition may require further qualifications [1] (pp. 37–38). These qualifications are, however, not germane for this article, because all phenomena considered here involve *h*, which is essential to the ultimate constitution of nature, if QM and QFT are correct, because *h* reflects the Planck scale, the ultimate scale of this constitution.

2 The concept of RWR and RWR interpretations were discussed in this author's previous works [1–4] (which also contain references to earlier works addressing the subject). These works will be cited here as appropriate. The present article follows them in outlining key concepts, such as complementarity, causality, reality, reality without realism and the arrow of events. I thought that providing these outlines would be beneficial to the reader, who would not need to consult these earlier works to follow my argument here concerning the relationships among complementarity, causality, and the arrow of events in quantum and quantum-like theories.

3 The designation ‘the Copenhagen interpretation’ requires even more qualifications as concerns whose interpretation it is and what are its features. Accordingly, I shall avoid this designation.

4 The probability density function specifies the probability of the random variables falling within a particular range of values, as opposed to taking on a single value. This probability is given by the integral of this variable's probability density function over this range. The probability density function is non-negative everywhere, and its integral over the entire space is equal to one.

5 Wave-particle complementarity, with which the concept of complementarity is often associated, had not played a significant, if any, role in Bohr's interpretation. His solution to the dilemma of whether quantum objects are particles or waves was that they were neither, any more than anything else—by definition in his ultimate, strong RWR, interpretation. When either ‘picture’ is used, it refers not to quantum objects but to either one or the other *set of discrete individual effects*, described classically, of the interactions between quantum objects and measuring instruments—particle-like, which may be individual or collective, or wave-like, which are always collective, composed of a large number of discrete individual effects. One needs of the order of 100 000 to observe the corresponding pattern of wave-like effects. A famous example is a manifold of ‘interference’ effects, composed of discrete traces of the collisions between the quantum objects considered and the screen in the double-slit experiment in the corresponding set-up, when both slits are open and there are no means to know through which slit each object has passed. Alternatively, one observes a discrete set of random, rather than interference-like, effects. While these *two sets of effects* are complementary (with the statistics for each correspondingly predicted by QM), the properties observed pertain to two mutually exclusive *sets* of discrete phenomena observed in instruments and not to any continuous phenomena or any continuous reality ultimately responsible for quantum phenomena. As an RWR-type reality, however, the reality ultimately responsible for quantum phenomena cannot be assumed to be discrete either.

6 Indeterminacy and randomness are sometimes distinguished by assuming that the first allows for probabilistic predictions, say, along one or another forms of Bayesian view, and the second does not. I assume the second view and hence shall speak of randomness, although most of my argument applies either way.

7 It is impossible to ascertain that an apparently random sequence of events is in fact random, rather than connected by some classical causal law, and there is no mathematical proof that any ‘random’ sequence is in fact random. The sequences of indeterminate events that allow for probabilistic predictions concerning them is a different matter, although there is no guarantee that any such sequence is not ultimately underlain by classically causal connections in the case of quantum phenomena. As explained, experimentally quantum phenomena only preclude determinism.

8 These concepts, or the present concepts of probabilistic and quantum causality, do have their genealogy in the preceding history of QM, beyond Heisenberg's and Bohr's view or those in quantum information theory, located, respectively, at the origin and the present moment of this history. One might even argue that from the inception of QM, its understanding and interpretation have been shadowed by the possibility or necessity of an alternative to classical causality that would nevertheless retain something akin to causal connections or correlations, probabilistic in nature, between events, versus merely random connections between them. Among the concepts defined that shaped this genealogy are the complementarity of coordination and the claim of (classical) causality in Bohr's 1927 Como lecture (quickly abandoned by him as this complementarity was, it remains important genealogically in considering quantum causality) [6] (v. 1, pp. 55), von Neumann and Birkhoff's concept of mathematical causation of quantum probabilistic predictions [17], and Heisenberg's concept of potentiality in his later work [7]. For an informed discussion of the last two concepts, via A. Shimony's concept of objective indefiniteness, and its own contribution to this problematic, see [18]. It is not possible to trace these concepts and connections among them here. The present concept of quantum causality and the arrow of events is more radical epistemologically (the concepts just mentioned are associated with realist views) by adopting a strong RWR interpretation and, especially, the Dirac postulate, and as such is more closely connected to the quantum-informational views of causality cited above.

9 J. Barbour adjusted his view on this point in his more recent work, in principle allowing for the arrow of time (he does consider the arrow of events), in connection with thermodynamics, within, however, a realist framework [20].

10 There are complexities in representing some of these combined symmetries, like CP, in the formalism of QFT, which I shall put aside here [21] (pp. 522–523).

11 The question of time in general is beyond my scope. See [23] for a review and references, which, however, does not consider anything akin to the RWR view and its potential impact on our understanding of time. The article limits itself at most to Kant's epistemology, which is not as radical. The famous debate on the subject between Einstein and H. Bergson will be put aside as well. Both positions involve subtleties (not always properly considered by either protagonist in critiquing the other) that would require a careful extended analysis.

12 The problematic just outlined is central to quantum Bayesianism (QBism), grounded in the subjective aspects (QBists would say ‘the subjective nature’) of quantum theory, leading the proponents of the approach to claiming the subjective nature not only (a) of our probabilistic predictions, but also (b) of the outcomes of quantum measurements. (See [25] for a clear exposition). While, as just explained, (a) is assumed here as well, (b) is not. In the present view, these outcomes are objective in the sense just outlined, specifically as unambiguously communicable. This assumption is correlative to the classical description of the observable parts of measuring instruments. QBism also assumes, correlatively to assuming both (a) and (b), that all objects or systems can be treated as quantum, while in the present view, some systems must be treated as classical by the Bohr postulate, and still others as relativistic. For a further discussion, see [26].

13 If one instantly repeats the same measurement the outcome will be, or is commonly (there are exceptions) assumed, to be the same. Apart, however, from the idealized nature of the assumption, I here refer to a very different repetition, that of the whole set-up from the beginning, *ab ovo* (e.g. [9], p. 161, [32], pp. 213–218, 335).

14 Obviously, singularity here or ‘thinking as singularity’ in the title of this section refers to a unique state and a unique course of reality of thought, and not to the mathematical sense of a singularity, say, as a point at which the mathematical object initially well-defined by means of an differential equation, is no longer well-defined.

15 The so-called many-worlds interpretation of QM does not affect this point, because this kind of material reality is still retained within each world involved, and there are no connections between these worlds.

16 This parallel has rarely been considered, apart from D'Ariano's recent work on the hard problem, as in his collaboration with F. Faggin cited above [34], which, however, does not address the RWR view of the situation.

17 It is true that one can use a technological device as an ‘agent’ initiating, or registering outcomes of, experiments. Any such device has, however, to be set up by a human agent, and it cannot make predictions, doing which is one of the characteristically features of human thinking and existence (although it is found in other animals).

18 For a compelling classical information-theoretical approach, see [45]. The analysis offered there is subtle and instructive, especially as concerns the role of metaphors, but is ultimately limited—unavoidably so, for fundamental reasons under discussion here, because of the role of qualia in literary works, including, and even especially, in metaphors, a role never considered in [45]. This role, I argue, defeats any classical-like informational (Kolmogorov) complexity, and requires, to begin with, the Q-L, non-Kolmogorovian, informational complexity, and I further argue, ultimately defeats any mathematical, even Q-like, handling. Poetry is about (mostly unformalizable) qualia and not merely mathematical-like (formalizable) linguistic play, found and sometimes important as it may be. This play is epistemologically Q-like insofar as how this play emerges, the reality that produced it, especially the unconscious, is RWR, but, unlike the 'play' of quantum phenomena by quantum theory, it cannot be predicted by mathematical means, or in any event, exceeds the capacity of such means. In fairness, the argument offered in [45] does suggest, in part against its own grain, the limitations of the informational-theoretical approach, still, however, remaining within the limits of the Kolmogorov complexity, without addressing quantum-informational complexity as non-Kolmogorovian, or quantum information theory. This is not surprising because Shannon information theory is classical-like, realist, or in any event naturally lends itself to realist interpretation.
